# Role of Absorbable Polysaccharide Hemostatic Powder in the Prevention of Complications After Axillary Lymph Node Dissection in Breast Cancer Patients: A Multicenter Retrospective Analysis

**DOI:** 10.3390/medicina61010079

**Published:** 2025-01-06

**Authors:** Simona Parisi, Francesco Saverio Lucido, Francesca Fisone, Roberto Ruggiero, Salvatore Tolone, Francesco Iovino, Antonio Santoriello, Federico Maria Mongardini, Maddalena Paolicelli, Ludovico Docimo, Claudio Gambardella

**Affiliations:** 1 Department of Advanced Since and Surgery, General, Mini-Invasive, Oncological and Obesity Surgery, Luigi Vanvitelli University of Campania, 80138 Naples, Italy; francescosaverio.lucido@unicampania.it (F.S.L.); francesca.fisone@unicampania.it (F.F.); roberto.ruggiero@unicampania.it (R.R.); francesco.iovino@unicampania.it (F.I.); claudio.gambardella2@unicampania.it (C.G.); 2 Department of Surgery, Breast Unit, Luigi Cobellis Hospital, 84078 Vallo della Lucania, Italy

**Keywords:** breast cancer, axillary lymph node dissection, seroma, absorbable polysaccharide hemostatic

## Abstract

*Background and Objectives*: Although breast surgery has undergone a drastic de-escalation in recent decades, axillary dissection is still indicated in some selected cases. Unfortunately, in 3–85% of cases, complications such as seroma formation occur, highlighting the need for more accurate hemostasis systems. The aim of this study is to evaluate the effectiveness of absorbable polysaccharide hemostatic such as Haemocer^TM^ in preventing postoperative seroma. *Materials and Methods*: Patients referred to two surgery centers for a diagnosed breast cancer and candidates for axillary lymph node dissection were retrospectively evaluated and included in Group A (treated with Haemocer^TM^) and B (control group). The primary endpoints were the drain output after 48 h, the daily amount just before the removal, and the duration of axillary drainage placement. Secondary endpoints included the presence of seroma at the ultrasound (US) follow-up, significant blood loss, hematoma, the duration of surgery, and postsurgical complications. *Results*: The drain output within 48 h was 196 ± 93 vs. 286 ± 38 mL in Groups A and B, respectively (*p* = 0.013). The daily output before the removal was 40 ± 7 mL in Group A and 47 ± 2 mL in Group B (*p* = 0.049). The duration of axillary drainage placement was shorter in the experimental group (7 ± 3 days) compared to the control group (10 ± 1 days) with a statistically significant difference (0.037). During the US follow-up, on days 7, 15, and 30, the number of patients affected by seroma and the volumes were lower in the experimental group. *Conclusions*: The adsorbable hemostatic powder proved to be effective both in reducing the volume of drained fluid postoperatively and in decreasing the number and volume of reported seromas during the US follow-up.

## 1. Introduction

Breast cancer (BC) is the most prevalent malignancy among women, with global estimates reporting 2.3 million new cases in 2020 [1]. The implementation of mammographic screening and advancements in innovative technologies such as tomosynthesis, elastography, contrast-enhanced spectral mammography (CESM), and artificial intelligence (AI)-based computer-aided detection have facilitated the early detection of BC. These advancements have supported the development of less invasive and more precise breast surgical techniques.

In recent years, the role of this latter has undergone radical changes, with the de-escalation occurring also in the axillary approach [2].

In the past, axillary lymph node dissection (ALND) was routinely performed to achieve local control of advanced disease. Since the 1990s, however, sentinel lymph node biopsy (SLNB) has replaced ALND as the gold standard [3]. However, despite ongoing clinical trials examining the necessity of ALND in cases of positive sentinel lymph nodes (SLN), ALND is still performed in cases of locally advanced disease not treated with medical therapy and in cases of metastatic SLN after neoadjuvant therapy [4].

One of the main reasons for the de-escalation of axillary surgery, along with the discovery of its marginal role in the systemic control of the disease, is the high rate of postoperative complications such as upper limb lymphedema, bleeding, and seroma. Seroma is a collection of fluid in the dead space of the axilla and is associated with a wide range of incidence, from 3% to 85% in different series [5]. It contributes to prolonged patient discomfort, manifesting as pain, delayed wound healing, skin flap necrosis, and infection. Additionally, late-onset seroma formation imposes an extra financial burden, as it often requires multiple outpatient visits for the aspiration of the accumulated fluid. Many studies have reported that seroma formation can delay the initiation of adjuvant therapy, thereby affecting oncological outcomes [6].

Several risk factors and pathophysiological mechanisms have been described but the pathogenesis of seroma formation is not yet fully understood. The dead space created by dissected tissue is initially filled with serous fluid. This fluid changes composition in the days following surgery, initially resembling lymph-like fluid mixed with blood clots, which indicates damage to lymphatic and blood vessels caused by the dissection. In fact, many authors suggested that the primary causes are cellular damage from thermal effects and incomplete obliteration of vessels and lymph ducts during dissection [7]. ALND has been replaced by sentinel lymph node biopsy (SLNB) in women with early clinically node-negative BC, offering precious information about axillary staging. ALND was still routinely performed in cases of positive sentinel lymph node (SLN), but in recent years, this paradigm has been questioned [4].

According to the most recent guidelines, ALND is performed only in selected cases, including patients with clinically node-positive BC in the primary surgery setting, those with residual disease following neoadjuvant chemotherapy (NACT) [8], women undergoing mastectomy with positive sentinel lymph nodes (SLNs) in the absence of radiation therapy, and patients with locally advanced inflammatory BC. Consequently, enhancing the efficacy and minimizing complications associated with axillary surgery remains clinically significant.

Several medical devices or agents, designed to ensure hemostasis, have been proposed in recent years. Fibrin sealants are highly concentrated solutions of fibrinogen and other cryoglobulins, which increase hemostasis and cell adherence. They were available as a form of glue or patch and were used in breast surgery and during ALND, reducing the seroma formation [9].

Resorbable polysaccharide powder (HaemoCer™; BioCer Entwicklungs-GmbH, Bayreuth, Germany) is a polymer of vegetable origin that can promote coagulation processes by increasing platelet concentration. The safety and efficacy of this hemostatic powder have been tested in various surgeries, gaining global acceptance [10,11,12].

The aim of the current study is to evaluate drain output after 24 and 48 h, the presence of seroma at ultrasound (US) scans or significant postoperative blood loss, and complications in patients treated with or without resorbable polysaccharide powder during axillary surgery.

## 2. Materials and Methods

### 2.1. Study Design

This study was reported according to the STROBE statement for cohort studies [13], and it was conducted according to the ethical principles stated in the Declaration of Helsinki. Written informed consent was obtained from all patients. It is a retrospective multicenter study aiming to compare the effectiveness of resorbable polysaccharide powder during axillary surgery in terms of drain output after 24 and 48 h, the presence of seroma at ultrasound (US) scans or significant postoperative blood loss, and complications.

### 2.2. Study Setting and Study Population

Patients referred to the General Surgery Division of the University of Campania “Luigi Vanvitelli” (Naples, Italy) and to the Breast Surgery department of Cobelli’s Hospital (Salerno, Italy) from 1 January 2017 to 31 December 2023 for a diagnosed BC and who were undergoing ALND were considered in the current study. Inclusion criteria included the following: presence of BC, indication for ALND after multidisciplinary discussion, and American Society of Anesthesiologists (ASA) physical status of grade ≤ IV [14].

Exclusion criteria were as follows: pregnancy, inflammatory BC distant metastases, autoimmune disease, previous BC or axillary surgery, previous thorax radiation treatments, chronic inflammation due to Hepatitis B or C virus infection, chronic gastritis, nephritis or gout, immune or hematological disease, heart failure, and the use of an anticoagulant drug. Moreover, skin-sparing and skin-nipple-sparing mastectomy followed by prosthesis or expander were excluded. Patients who received surgery with any coagulative devices were not included in the study.

Patients who underwent neoadjuvant treatments were excluded from the study due to the potential bias caused by fibrotic outcomes on the breast and axilla. Therefore, patients with axillary lymph node involvement who refused systemic therapy or had contraindications due to comorbidities were enrolled. Additionally, some cases of patients with luminal breast cancer were included, as the multidisciplinary oncology group did not recommend neoadjuvant therapy for them, in accordance with the latest NCCN guidelines (Version 3.2024) [15].

All participants underwent a preoperative blood sample, mammography, breast and axillary US, and a core biopsy of the breast lesion in the 30 days prior to surgery. After referral for surgery, each patient received a detailed explanation of the procedure from the medical staff and had to sign a personalized informed consent form. All the procedures were performed by the same experienced surgeons.

Clinical data were collected in an electronic database and retrospectively analyzed. Patients were divided into two groups: Group A, patients receiving ALND with the application of the product (a 3 g package), and Group B, patients receiving conventional ALND, which was considered the control group. The application of the hemostatic powder in Group A was not related to controlling the local hemostases but was routinely performed in one of the surgical divisions.

Clinical and demographical parameters (age, sex, BMI, number of dissected lymph nodes, intraoperative blood loss, volume of drainage) were evaluated, as well as the postoperative outcomes (day of discharge, rates of complications, onset of seroma or hemorrhage, reoperation). The follow-up consisted of regular clinical and instrumental examinations of the axilla until the fortieth day following discharge.

### 2.3. Surgical Technique

Patient positioning that allows maximum exposure is essential. Therefore, the patient was positioned in the supine position, with the lateral chest wall placed at level with the edge of the table. The arm was draped separately and placed on an arm board, abducted to 90°. Rotation of the patient away from the seated operator provides good vision towards the apex of the axilla. An incision was made up the skin projection and parallel to the lateral edge of the pectoral muscle, approximately 2 cm from it (extension of the ellipsoid excision when associated with mastectomy or upper-lateral quadrantectomy, separate excision when associated with other quadrantectomies or tumorectomies), near the axillary fossa was performed just below the hair-bearing area of the axilla. The limits of incision are the anterior and posterior axillary folds, and a better cosmetic result is obtained if these are not crossed. The axillary dissection was carried out just after the breast procedure. Immediately after opening the clavipectoral fascia and entering the adipose tissue, the axillary vein, the vessels, and the thoracodorsal nerve are identified and preserved. ALND was carried out to remove all visible axillary lymph nodes (ALNs) in I and II Berg’s levels, preserving the intercostal nerves wherever possible. At the end of every procedure, a closed suction Redon drain was placed in the axilla cavity. Both groups received ALND with an electrosurgical knife.

### 2.4. Outcome Measures

The mean surgery time was reported in minutes and median hospitalization was reported in days. After surgery, drain output was recorded daily and the drain was removed when the output was less than 50 mL/day. The mean drainage output was evaluated in milliliters. The mean drainage output was evaluated in milliliters. The presence of any other postoperative complications such as hematoma formation, wound infection, pain, and bleeding were signed daily in the medical records and were expressed as the number of cases and as a percentage. Axillary ultrasonography was performed 48 h after the surgery, before the drain removal, and on days 7, 15, and 30. High-quality US-ES images were acquired using Aixplorer^®^ Mach 30 (Supersonic Imagine, Aix-en-Provence, France) with a linear probe L18-5 (centered at 10 MHz). Each patient was positioned supine with the arm raised above the head, and the entire axillary region was scanned. The examination was conducted by a sonographer with almost 10 years of experience in breast and axillary US. The presence of a seroma was reported with the main 3 measures (longitudinal, transverse, and anteroposterior diameters) expressed in millimeters, and the volume was calculated with the ellipsoid formula [16].

### 2.5. Study Endpoints

The primary endpoint was the drain output (mL) after 48 h and the daily amount just before the removal. Moreover, the duration of axillary drainage placement was also included in the primary aims. Secondary endpoints included the presence of seroma at the US scans following the drain removal and at days 7, 15, and 30, significant blood loss (if the patient needed to return to the operating room), hematoma, and postsurgical complications. The main indications for reoperation were bleeding with compression, acute anemia, and the presence of more than 400 cc of bright red blood in the drainage.

### 2.6. Statistical Analysis

Continuous variables were presented as mean and standard deviation, while categorical variables were reported as frequencies (number of cases). The study population was divided into two groups: Group A, comprising patients treated with the application of hemostatic powder and aspiration drainage, and Group B, consisting of patients treated with aspiration drainage alone, serving as the control group. Independent sample t-tests were conducted to compare continuous variables, including age, length of hospital stay, BMI, number of axillary lymph nodes, operation time, and drainage and seroma volumes. A *p*-value < 0.05 was considered indicative of statistical significance. Statistical analyses were performed using SPSS software, version 23 (SPSS©, Chicago, IL, USA).

## 3. Results

From 1 January 2017 to 31 December 2023, 2357 patients with breast disease were referred to two high-volume centers. Among them, 409 subjects presented the inclusion criteria and were enrolled in the study. In Group A (Hemostatic Powder), 221 patients were included, while Group B (Standard Hemostasis—Control Group) was composed of 188 patients. All the participants were women. The mean age was 62.1 ± 4.5 years in Group A and 60.6 ± 7.8 years in Group B (*p*-value = 0.674).

Demographic and clinical data are detailed in Table 1. No significant differences were observed in demographic characteristics between the two groups. In Group A, the mean number of dissected lymph nodes was 14.8 ± 2.7, while in Group B, it was 13.9 ± 3.4, without significant differences (*p* = 0.126). Radical mastectomy was performed in 23 patients (10.4%) in Group A and 16 (8.5%) in Group B (*p* = 0.515); quadrantectomies were the most performed surgery in both the groups (144 in Group A (65.1%) and 131 in Group B (69.7%), *p* = 0.331). Moreover, 54 patients in Group A received a tumorectomy (24.5%) vs. 41 (21.8%) in Group B (*p* = 0.530). The duration of the surgical procedure was shorter in Group A than in the control group, but the difference was not statistically different (76.5 ± 16.5 vs. 77.6 ± 14.7, *p* = 0.346) (Table 2).

### 3.1. Primary Endpoints

**Figure 1:**
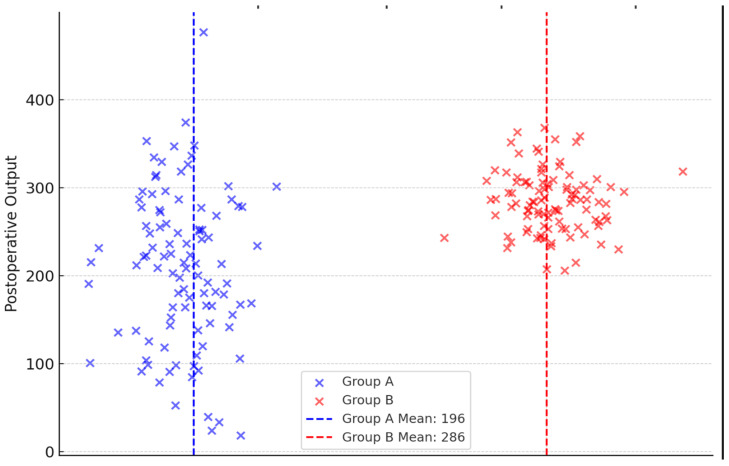
Dot plot: postoperative drain output.

The drain output within 48 h was 196 ± 93 vs. 286 ± 38 mL in Groups A and B, respectively, with a statistically significant difference (*p* = 0.013) (Figure 1). The daily output before the removal was 40 ± 7 mL in Group A and 47 ± 2 mL in the control group (*p* = 0.049). The duration of axillary drainage placement was shorter in the experimental group (7 ± 3 days) compared to the control group (10 ± 1 days), with a statistically significant difference (*p* = 0.037).

### 3.2. Secondary Endpoints

Following the drainage removal, during the follow-up, at day 7, 29 patients (13.1%) were affected by axillary seroma vs. 44 (23.4%) in Group B (*p* = 0.010). At day 15, 18 (8.1%) people vs. 34 (18.1%) reported a measurable seroma on ultrasound in Groups A and B, respectively, with a statistically significant difference (*p* = 0.003). After 30 days from the surgery, only 10 participants in Group A (4.5%) and 18 (9.6%) in Group B still had a residual seroma (*p* = 0.002).

A further aim of the study was to evaluate the seroma sizes at US. In Group A, the seroma volume was 12.6 cm^3^ vs. 17.2 cm^3^ in the control group (*p* = 0.190). At day 15, the volumes were 4.5 cm^3^ and 8.7 cm^3^, with *p*-value = 0.002.

Significant blood loss occurred in one (0.5%) patient of Group A and two (1%) patients of Group B (*p* = 0.470), and all the patients returned to the operative room. The length of hospital stay did not differ between the two groups examined (2.2 ± 0.4 in Group A and 2.5 ± 0.5 in Group B, *p* = 0.334).

No significant difference was reported about any wound infections (7 cases in Group A (3.1%) vs. 11 (5.9%) in Group B, *p* = 0.187) and axillary pain (58 people in experimental (26.2%) and 63 in the control group (33.5%), *p* = 0.183).

## 4. Discussion

Since the 18th century, axillary dissection has been considered an integral part of BC therapy because the regional lymph nodes represented the point of spread via the lymphatics to distant sites [4,5,6,7,8,9,10,11,12,13,14,15,16,17]. Therefore, Halsted proposed the radical mastectomy, including the removal of axillary lymph nodes, as the standard treatment for patients with breast cancer [18]. Starting from the 1900s, there has been a strong de-escalation in breast surgery, which subsequently similar impact on the management of the axilla. SLNB became the current practice standard of care for women with early-stage BC and non-suspected lymph nodes. However, there has been recent debate about the necessity of performing ALND even in cases of micrometastatic SLNs [19]. According to the ACOSOG Z0011 (Alliance) randomized clinical trial, findings did not support the routine use of ALND in women with T1/T2 BC, without palpable axillary adenopathy, and one or two sentinel lymph nodes containing metastases [20]. These results were confirmed by the AMAROS trial, highlighting that ALND and axillary radiotherapy after a positive SLN provide a comparable axillary control for patients with T1-2 BC, in the absence of axillary suspect. Radiotherapy was associated with significantly less morbidity, proving to be more advantageous than surgery [21]. In the post-neoadjuvant setting, many studies showed that SLN was also effective in cases of negativized nodes [22].

However, the 2019 St. Gallen consensus conference established that SLNB was adequate if at least three or more negative sentinel nodes were detected and examined. Moreover, the Panel recommended that patients with a clinically positive axilla or with macrometastases identified in SLN after neoadjuvant therapy undergo ALND. The Panel was divided over whether residual micrometastatic lymph node involvement needed completion dissection after NAC. The conclusion was in favor of the execution of ALND unless regional nodal irradiation was not planned [23]. Therefore, although less frequent, lymphadenectomies are still indicated in global guidelines, and improvements in surgical techniques and the reduction of complications are highly desired. In the literature, two major lines of investigation are reported regarding the reduction of postoperative seromas: surgical energy devices and hemostatic agents.

Regarding surgical instruments, various surgical energy devices are proposed for ALND, and many studies aimed to compare the different performances in breast and axillary surgery, investigating the intraoperative and postoperative outcomes, especially seroma formation. A randomized controlled trial compared the outcome of patients undergoing breast surgery and axillary dissection using either standard scalpel blades, scissors, ligations, and electrocautery or the ultrasound scalpel only, verifying that the use of the harmonic scalpel was shown to reduce the magnitude of seromas in the axilla and hospitalization stay [24].

Gambardella et al. also performed a comparative study including innovative devices such as Thunderbeat. Electrocautery, Harmonic Scalpel, LigaSure, and Thunderbeat performances were evaluated, concluding that Thunderbeat was superior in terms of reduction of intraoperative blood loss and postoperative drainage output. Moreover, it was associated with a substantial reduction in postoperative seroma incidence [25]. A meta-analysis synthesized the current evidence about surgical energy. Compared to the conventional techniques, ultrasonic coagulation devices likely reduced seroma (risk ratio [RR], 0.61; 95% credible interval [CrI], 0.49–0.73), the drained fluid volume (mean difference [MD], −313 mL; 95% CrI, −496 to −130), and drainage duration (MD, −1.79 days; 95% CrI, −2.91 to −0.66). The authors concluded that elettropolar devices might have had little effect on seroma, the drained fluid volume, and drainage duration compared to conventional techniques [26].

Several hemostatic systems were also studied and proposed in numerous studies, with controversial results, such as fibrin glue spray. Some authors proposed bovine thrombin spray on seroma formation following breast surgery with axillary dissection, but no significant advantages were identified [27]. Another recent study evaluated patients treated with tranexamic acid compared to a control group, showing a significantly lower axillary drainage in the experimental group (440 mL vs. 715.5 mL, *p* = 0.003) with earlier removal of the drain (8 vs. 11 days, *p* = 0.046). Seroma formation (19.1% vs. 32.6%, *p* = 0.13) and wound-related infection (4.3% vs. 8.7%, *p* = 0.43) were not significantly different.

To the best of our knowledge, this study is the first to analyze the impact of a hemostatic resorbable polysaccharide powder after ALND, also adopting an US follow-up. Our experiences are consistent with the report by other groups and are very encouraging in terms of both the reduction in the number of postoperative seromas and their sizes. Furthermore, we must emphasize that we have detected the presence of seroma even after the removal of the drainage, with US scans, providing more detailed information during follow-up. This could partly explain the different results compared to other studies. Therefore, we aim to verify the results in a future study, distinguishing between lumpectomies and mastectomies.

A previous study highlighted the advantages of using hemostatic powder in thyroid surgery, demonstrating its efficacy as a hemostatic agent in reducing postoperative drainage output and minimizing complications such as neck hematoma and seroma, thereby enhancing postoperative patient comfort [10]. More recently, a randomized controlled trial evaluated the use of an absorbable polysaccharide hemostatic agent during surgery. In this trial, 68 patients were randomized to receive the hemostatic agent applied to the wound site, while 68 patients in the control group did not receive any hemostatic agent. Contrary to expectations, patients in the intervention group exhibited significantly higher drainage output volumes compared to the control group, with median volumes of 85 mL (IQR 46.25–110) versus 50 mL (IQR 30–75), respectively (*p* =  0.003). The study concluded that the intraoperative application of a polysaccharide hemostatic agent during breast-conserving surgery did not reduce postoperative fluid production [28]. However, these results are inconsistent with experiences in thyroid and renal surgery, as reported in the study published by Burghuber et al. They found that out of 147 patients enrolled in the study treated with the hemostatic powder, 15 developed lymphoceles, which represents a rate of 10.2%; (95% CI: 6.3–16.2%), and compared to the expected occurrence, this was significantly lower (*p* = 0.003) [29]. Some authors have demonstrated that a predictive factor for seroma formation is the size of the excised tissue [28,30]. Although our focus was on axillary lymphadenectomy, we cannot exclude that total mastectomy may have had a different impact compared to lumpectomies, especially if not in continuity with the axillary cavity.

The paper has some limitations to address: the retrospective nature of the study and the limited sample size. Moreover, we chose to exclude patients undergoing reconstructive surgery from the study to avoid bias related to inflammatory reactions from foreign bodies. However, we included in the study patients who underwent minimally invasive breast surgeries as well as radical mastectomies.

The total duration of the surgery was considered, including the time for breast surgery. Although this data does not exclusively reflect the axillary dissection, no significant differences were found between the procedures in a sample of 409 patients.

## 5. Conclusions

The use of absorbable polysaccharide hemostatic powder was effective in the prevention of complications after axillary lymph node dissection in breast cancer patients. It reduced both the volume of drained fluid postoperatively and the number and volume of reported seromas during the US follow-up, without showing sequelae or prolonging the length of surgery. Further larger comparative studies are needed to address this issue.

## Figures and Tables

**Table 1:** The baseline clinical and demographic features in both groups.

	Group A (n = 221) Hemostatic Powder	Group B (n = 188) Control Group	*p*-Value
**Age** (years) °	62.1 ± 4.5	60.6 ± 7.8	0.674
**BMI (kg/m^2^)**	23.7 ± 3.0	24.0 ± 1.8	0.834
**Hypertension**	75 (33.9%)	61 (32.4%)	0.725
**Smoking**	61 (27.6%)	57 (30.3%)	0.545
**Number of lymph nodes °**	14.8 ± 2.7	13.9 ± 3.4	0.126
**Mastectomies °**	23 (10.4%)	16 (8.5%)	0.515
**Quadrantectomies °**	144 (65.1%)	131 (69.7%)	0.331
**Tumorectomies °**	54 (24.5%)	41 (21.8%)	0.530

° Median and standard deviation; BMI, body mass index.

**Table 2:** Study outcomes.

	Group A (n = 221) Hemostatic Powder	Group B (n = 188) Control Group	*p*-Value
**Drain output after 48 h (mL)** °	196 ± 93	286 ± 38	0.045 *
**Daily drain output before the removal (mL)** °	40 ± 7	47 ± 2	0.049 *
**Duration of axillary drainage placement (days)**	7 ± 3	10 ± 1	0.037 *
**Patient with axillary seroma at US:**			
**-day 7**	29 (13.1%)	44 (23.4%)	0.010 *
**-day 15**	18 (8.1%)	34 (18.1%)	0.003 *
**-day 30**	10 (4.5%)	18 (9.6%)	0.044 *
**Axillary seroma volume at US:**			
**-day 7**	12.6	17.2	0.019
**-day 15**	4.5	8.7	0.018 *
**-day 30**	1.6	4.8	0.002 *
**Significant blood loss (%)**	1 (0.5%)	2 (1%)	0.470
**Day of discharge**	2.2 ± 0.4	2.5 ± 0.5	0.334
**Surgical site infection**	7 (3.1%)	11 (5.9%)	0.187
**Axillary pain**	58 (26.2%)	63 (33.5%)	0.108
**Operation time (min)** °	76.5 ± 16.5	77.6 ± 14.7	0.346

* Statistically significant value; ° Median and standard deviation.

## Data Availability

The original contributions presented in this study are included in the article. Further inquiries can be directed to the corresponding author.
